# Supplementary material for: Acceptability of a community-embedded intervention for improving adolescent sexual and reproductive health in south-east Nigeria: A qualitative study
Source: PLoS One. 2023 Dec 14;18(12):e0295762. doi: 10.1371/journal.pone.0295762 (PMC10721091; doi:10.1371/journal.pone.0295762)
Supplement: S1 File — (DOCX) [file pone.0295762.s001.docx]

**ADOLESCENTS AND COMMUNITY MEMBERS**

**Name:** Affective attitude

**Description:** Any reference to the effectiveness of the intervention. How relevant, important, beneficial, helpful or necessary the intervention was. Participants responsiveness to SRH intervention eg enthusiastic, eager to learn

<Files\\A FOCUS GOUP DISCUSSION WITH FEMALE PARENTS IN NWOFE COMMUNITY> - § 14 references coded [13.92% Coverage]

Reference 1 - 0.77% Coverage

The campaign is beneficial to all of us in this community, it reduced the rate at which girls are pregnant in this community. It also made the parents to know how to discuss with their children

Reference 2 - 0.45% Coverage

It helped parents to know how best to talk to their adolescents to tell them what they are supposed to know or do

Reference 3 - 0.79% Coverage

it is of great benefit to the children because they have knowledge of all part of their bodies and what they represent and know how to take care of sensitive parts of their bodies to avoid problems.

Reference 4 - 0.66% Coverage

The campaign was beneficial because some adolescents do not know how and the important of keeping themselves clean but the campaign exposed them to all these things

Reference 5 - 0.69% Coverage

It helped us on the side of our health, we have being hearing about condom but do not understand it, but this campaign has made it clearer for us to understand its importance

Reference 6 - 0.47% Coverage

I learnt so much about adolescent stage, they were taught how they can prepare themselves and be successful in life

Reference 7 - 0.49% Coverage

It was very relevant because if the campaign did not take place, we would have been leaving in ignorance and ignorant kills,

Reference 8 - 1.50% Coverage

The campaign is of great importance because adolescent age is specifically known as a vital stage in which if they are not properly handled and they are not able to control themselves, the persons future will be shattered, so the campaign is very importance and should be as often as possible because it guides the adolescents on how they can control themselves in the future

Reference 9 - 1.94% Coverage

The campaign is very important because there is no child that will not pass through the adolescent stage even the tender ones, we the adults have seen the importance. In the time pasts, you will not see anybody that will come to you to discuss SRH, to guide and advice you to put yourself together but now, the freedom is here, we can freely discuss with our children about SHR, we discuss it in the homes and classes to inform them about the advantages and disadvantages of everything.

Reference 10 - 0.25% Coverage

It is appropriate because it has change some of our behaviors

Reference 11 - 1.75% Coverage

It is appropriate because it has exposed us to many things that we do not know before, it helped in exposing the adolescents to the truth, it is true that they know about some of these things but they might be getting the information from wrong sources, with this campaign they have known the truth, if they happen to get information about reproductive health from any other source, they will weigh it then decide on the right one to chose

Reference 12 - 0.72% Coverage

It is very important to the extent that we would put more effort to continue with the campaign, our children understand all those things and are obeying the content of the campaign.

Reference 13 - 0.34% Coverage

It is important because the campaign changed many things in the lives of our children

Reference 14 - 3.10% Coverage

The campaign is in line with my roles and responsibilities as a mother because if you are enlightened to an extent and people notice that you discuss reproductive health with your children, they will begin to see you as if you are going extra mile to discuss those things, you will be saying those things to your children in secret so that neighbors will not hear what you are telling them but now, you can freely talk to your children about SRH to your children without missing words, you will boldly say it without hiding from people’s blame knowing very well that your children will definitely tell other people’s children what you have discussed with them and they will see you as an irresponsible mother but now the children can freely discuss what their mother told them

<Files\\FGD WITH ADOLESCENT BOYS IN AGALEGU COMMUNITY> - § 10 references coded [10.13% Coverage]

Reference 1 - 1.50% Coverage

I am very happy about the campaign, it was done in 2020 and there are great and positive improvements in our lives, young girls and boys in this community stopped moving about at 8:00 at night, we are behaving very well now and things have changed

Reference 2 - 1.16% Coverage

It is a good one, they did very well by coming becuase the teaching changed many things, if not for the campaign, many young girls would have been pregnant by now, the campaign is a good one

Reference 3 - 0.68% Coverage

It is relevant because the teaching changed our lives, we stopped doing some of the things we were doing before

Reference 4 - 2.15% Coverage

Yes, it is important to discuss those things among adolescents because if you fail to discuss the right things with us, we will indulge in something bad which will affect us later in future but teaching all these will help us to do good things. It is good to teach us so that wherever we find themselves, they will continue to do good and teach others too.

Reference 5 - 0.44% Coverage

I am very happy since I joined this group and since we started teaching

Reference 6 - 1.09% Coverage

It gladdens my heart because some of the bad things that were going on have stopped, some young boys used to pregnant young girls in this community but it is no more happening now

Reference 7 - 0.69% Coverage

I am very happy now because girls no longer move about at night, we listen and obey what our parents tells us to do

Reference 8 - 0.75% Coverage

I am happy because the rate of unwanted pregnancy has reduced in this community. I am happy that I teach my friends about SRH

Reference 9 - 0.23% Coverage

I am happy that I belong to this group

Reference 10 - 1.42% Coverage

I am happy to be part of the people that is teaching SRH to adolescents because if we I did not tell them about it, they will not know about it. Each time I tell people not to continue in any life style they usually adhere to my advice

<Files\\FGD WITH ADOLESCENT GIRLS IN NWOFE COMMUNITY> - § 14 references coded [11.82% Coverage]

Reference 1 - 0.34% Coverage

P4. Yes, because it has been helpful to us

Reference 2 - 1.96% Coverage

P4. It is important because our lives are better as we obey instructions that were given to us during the campaign. Many of the young girls were getting pregnant before now, but it has reduced very well, we learnt many things from the campaign.

Reference 3 - 0.77% Coverage

We learnt many things that is helpful to us because unwanted pregnancy reduced in this community

Reference 4 - 0.36% Coverage

It helped us to do what is good for ourselves

Reference 5 - 0.31% Coverage

P5. You are right, it helped us so much

Reference 6 - 0.81% Coverage

P3. It helped us because you don’t need to ask someone since the messages are being spread of gospel

Reference 7 - 1.50% Coverage

P3. I like it as it is an opportunity for me to learn many thing for myself, it also help me to talk to my friends and others so that we know how best to take decision when anything arise

Reference 8 - 0.42% Coverage

P1. Yes, it is useful to me and made me to feel well

Reference 9 - 0.21% Coverage

P2. It made me to be happy

Reference 10 - 1.14% Coverage

P3. I feel happy because I am a member of this group because of the things we were taught many things that we can teach our sisters and parent

Reference 11 - 0.74% Coverage

P4. It is use for us because we learnt many things, even the one we will teach our children

Reference 12 - 1.27% Coverage

P3. It made me to be happy and proud and I have learn how to talk to my siblings and other people around me so as not to fall a victim of what they don’t know

Reference 13 - 0.98% Coverage

P5. It made me happy because anytime I want to do those things that we used to do before, I will remember it and withdraw

Reference 14 - 1.00% Coverage

P6. It made me to be happy because I learnt a lot of things that I will teach those that are growing up, even my own children

<Files\\FGD WITH ADOLESCENTS BOYS IN NWOFE COMMUNITY> - § 8 references coded [14.21% Coverage]

Reference 1 - 3.05% Coverage

P3. It is very good on the side of boys and girls, before now, many of our girls enter into the trouble of unwanted and teenage pregnancy but since the commencement of the campaign in the community, teenage pregnancy reduced drastically.

Reference 2 - 1.24% Coverage

P1. It is good because I have stopped leaving those lives that attracts quarrels from my parents

Reference 3 - 2.27% Coverage

P6. The teaching helped us to avoid unwanted pregnancy

P5. It is good because we learnt that it is not good to use condom when you want to have sexual intercourse with a girl

Reference 4 - 0.24% Coverage

P3. It is important

Reference 5 - 1.23% Coverage

P4. It is important because sexually transmitted infections are no more common in this community

Reference 6 - 2.11% Coverage

P2. It is important because before now, you will see many girls with unwanted pregnancy but now you will hardly see them or hear that a particular girl is pregnant

Reference 7 - 3.78% Coverage

Yes [How has it helped you people in getting correct information] because there are people that find it difficult to go to the nurses to get correct information instead they will prefer to get the needed information from their friends or take drugs without prescriptions from the health workers

Reference 8 - 0.28% Coverage

P4. It is a good thing

<Files\\FGD WITH ADOLESCENTS GIRLS IN AGALEGU COMMUNITY> - § 10 references coded [8.46% Coverage]

Reference 1 - 1.17% Coverage

P6. It is good, they thought us that it is wrong for boys and girls to be having unsafe sexual intercourse

Reference 2 - 0.55% Coverage

P3. It is good for us not to get unwanted pregnancy

Reference 3 - 0.50% Coverage

P2. It is good for us to take care of ourselves

Reference 4 - 0.20% Coverage

P2. Yes, it is good

Reference 5 - 1.72% Coverage

P5. Yes, it is important because we were taught how we will take care of ourselves to avoid contraction of HIV/AIDS and syphilis and how to avoid other diseases

Reference 6 - 0.91% Coverage

P4. It is important, it helps us not be get unwanted pregnancy or to commit abortion

Reference 7 - 1.38% Coverage

P6. Yes, it was relevant because it will make us to avoid unwanted pregnancy and helps us not to be exposing our body and be neat

Reference 8 - 0.95% Coverage

P5. Yes, I am happy because they taught us to avoid unwanted pregnancy and how to be neat

Reference 9 - 0.53% Coverage

P6. It made me to know what I do not know before

Reference 10 - 0.55% Coverage

P2. For me, it made me to know about boys and girls

<Files\\FGD WITH FEMALE COMMUNITY LEADERS IN AGALEGU COMMUNITY IN IKWO> - § 8 references coded [5.06% Coverage]

Reference 1 - 0.13% Coverage

It is a fruitful thing,

Reference 2 - 0.38% Coverage

It is good that we are telling our children about sexual intercourse, if

Reference 3 - 0.16% Coverage

The campaign is a good thing,

Reference 4 - 0.11% Coverage

It is a good thing,

Reference 5 - 1.93% Coverage

It is very relevant because our children learn many good things from SRH campaign, they have known what they are supposed to do and what they are not supposed to do

P2. It is relevant because it is an eye opener to all of us, we have known the good and the bad

P1. It is relevant because it made them to see the need to obey what their parents are telling them

Reference 6 - 0.84% Coverage

P1. It is appropriate and they are learning what we are teaching them

P2. It is appropriate as it beard good fruit among the adolescents

P5. It is fruitful

Reference 7 - 1.17% Coverage

P4. It helped in a great way because sexual intercourse among the adolescents stopped since the campaign started. Before now, we send out any of our daughters who get unwanted pregnancy but now we don’t send them out

Reference 8 - 0.35% Coverage

P3. It is a good program because it is good for me and my family

<Files\\FGD WITH MALE COMMUNITY LEADERS IN AGALAGU AMAGU, IKWO LGA> - § 8 references coded [8.09% Coverage]

Reference 1 - 2.90% Coverage

My view on the small group awareness in this community is that it has helped our adolescents in the community to the dos and Don’ts in terms of their sexual and reproductive health rights. The adolescents have been putting what they learnt into practice because of the ways and manners they now behave amongst their peers and elderly ones in the community. The truth is that the awareness has been bearing good fruits in the community and we know that the benefits will continue to be evident in the community. We are thanking the initiators and implementers of the intervention because it has really changed a lot for adolescents positively including their dressings and other indecencies amongst the adolescents in the community.

Reference 2 - 0.54% Coverage

In fact I thank the government that came up with this initiative having made available this intervention for adolescents to benefit from,

Reference 3 - 1.09% Coverage

In the first place the awareness intervention has really done much good to our adolescents in this community because before now many of our adolescents usually opt for early marriage without minding the implications but now it is no more due to the awareness intervention.

Reference 4 - 0.91% Coverage

From my view, I feel it's relevant because if our children were allowed to continue the way they were it should have lead to mess and rotten society for us but awareness intervention has gone a long way transformed their lives.

Reference 5 - 0.82% Coverage

Yes it's very appropriate because had it been you did not come with the awareness campaign on our adolescents things should have gotten very bad, there have been so many changes since the awareness campaign,

Reference 6 - 0.73% Coverage

Mine is to add to what the last speaker said, the awareness intervention is appropriate because it has changed a lot positively in the lifestyles and behaviours of our adolescents and

Reference 7 - 0.58% Coverage

the summary of what I want say is that the coming of your intervention has yield much positive fruits in the lives of our youths in this community.

Reference 8 - 0.52% Coverage

The teaching helps them to know that the right time for sexual intercourse is when the person is married and ready to have children,

<Files\\FGD WITH MALE PARENTS IN NWOFE AGBAJA COMMUNITY, IZZI LGA> - § 8 references coded [8.01% Coverage]

Reference 1 - 1.42% Coverage

The program is a good one because it helped and opened the eyes of parents and adolescent towards these things so that people can easily understand the advantages and disadvantages of all these things whenever you talk to them.

Reference 2 - 1.39% Coverage

It helped very well for better understanding of some of those things we use to hide before, the adolescents can know that knowledge of SRH can protect them from infection and can also protect them from committing atrocities

Reference 3 - 0.87% Coverage

They are very relevant because the information they gave out helped in controlling negative sexual behaviours and diseases among adolescents

Reference 4 - 0.90% Coverage

The benefit is quite enormous, it helped in many ways to eradicate STDs and unwanted pregnancy and most of the sexual activities and other things

Reference 5 - 1.03% Coverage

P1. Yes it is appropriate because it prevent many diseases and unwanted pregnancies

P2. It is appropriate and needs to be sustained due to the impact it has created

Reference 6 - 0.79% Coverage

P2. Yes, the community leaders commended the program so much the day they were invited here because they message is everywhere

Reference 7 - 1.49% Coverage

It fits into our teachings because you can’t just call and discuss with your child on certain issues not until after the campaign, we became disposed and free to discuss sensitive issues with our children, it yielded fruits since that time

Reference 8 - 0.12% Coverage

It is very useful,

<Files\\FGD_Adolescents In School_Female_Afikpo South> - § 12 references coded [7.77% Coverage]

Reference 1 - 0.78% Coverage

My view about the school health that is taking place in our school is that it has helped young girls to be educated about sexual harassment and how to report it if we are harassed sexually by anybody.

Reference 2 - 0.78% Coverage

I am ‘seeing this s health club as ‘a very interesting one that help we young girls to express ourselves, about what we come across in our homes and school; of which we do meet our G and C guidance.

Reference 3 - 0.49% Coverage

It’s very important to us especially in our school. It helps us to take good care of ourselves during our menstrual cycles.

Reference 4 - 0.43% Coverage

It’s important to us because it helps us to promote the adoption of sexual health behaviours and reproduction

Reference 5 - 0.70% Coverage

Its relevant in our school because it has changed so many teenagers because some of them had not been thinking well but this club came into our school it has changed our lives.

Reference 6 - 0.16% Coverage

The school health club helped very well

Reference 7 - 0.87% Coverage

How it helped is that sometimes teenagers want to know the reason why we should not engage in sexual behavior, and the school health club has given answers to our questions to know the reason and dangers why we should not.

Reference 8 - 0.52% Coverage

The school health club has provided enough information for us to know the dangers of teenage pregnancy and for us not to contact STD

Reference 9 - 1.55% Coverage

P7 – Am happy about it because I learnt a lot of things in it.

P6 – Am so glad and excited to be in it because as a teenager it has opened my eye.

P8 – Am very happy about it because it has made those of us members to know much about it because there are a lot of thing we learn that we do teach them that they are not aware of.

P9 – Am very happy about it because it has made me to be bold.

Reference 10 - 0.13% Coverage

P9 – it does make me feel relevant

Reference 11 - 1.03% Coverage

P5 – It has made me to be so valued because in a situation of menstruation cycle I now know how to take care of myself.

P8 – It has made me valued because in the past I hardly stand firm and make decision but now I can stand firm and made decision on my own.

Reference 12 - 0.33% Coverage

P5 – It help students to know the risk in sexual intercourse and teenage pregnancy.

<Files\\IDI WITH VICE PRINCIPAL, EZZA HIGH SCHOOL, EZZA SOUTH> - § 5 references coded [5.01% Coverage]

Reference 1 - 1.32% Coverage

Thank you very much. I participated in the training and my impression about it is that the intervention is necessary, it worthwhile because it impacted a lot on the lives of our students, the adolescents we handle here and most of who participated in the activity gained a lot.

Reference 2 - 0.30% Coverage

It is really relevant, it's important and strategic, we need it.

Reference 3 - 0.23% Coverage

It is appropriate, it should be deepened rather.

Reference 4 - 0.79% Coverage

Why do you think it is appropriate?

R. It is because the targeted audience show it. They show that they have learnt what is good and they long for the continuation.

Reference 5 - 2.36% Coverage

like I have told you that those children that took part in the training have been asking me "when will that training come again?" mothers and parents have been asking me, that anytime the team is coming again I should not fail to tell them so that their children will attend. Many of them have come out from secondary school, some are undergraduates and they keeping saying let the information come early so that they will send to them and that is how it is within the vicinity, it is fruitful.

HEALTH WORKERS

**Name:** Affective attitude

**Description:** Any reference to how the sexual and reproductive health intervention (SRHI) was valued as being relevant, important, beneficial, helpful or necessary the intervention was. Participants responsiveness to SRH intervention eg enthusiastic, eager to learn

<Files\\AN IDI WITH IZZI FOCAL PERSON> - § 2 references coded [4.18% Coverage]

Reference 1 - 2.28% Coverage

from my own view, it [the intervention] was very good, it went well, all the training we did at that time was good and we step down the training to other OIC’s, the CHEWS and Patent Medicine Vendors, the program makes the adolescent girls not to get pregnant again,

Reference 2 - 1.90% Coverage

Is very relevant and we are seeing it manifesting, because we see the adolescents coming to collect contraceptives and with that, if not that, you would have been seeing many girls getting pregnant, but now, it has reduced

<Files\\AN IDI WITH IZZI L.G.A ADMINSTRATIVE SECRETARY> - § 2 references coded [2.24% Coverage]

Reference 1 - 1.38% Coverage

my views is, I will first of all, appreciate the efforts of this partner, it helps us to improve on the health needs of adolescents on sexual reproductive services, it is improving,

Reference 2 - 0.86% Coverage

is very relevance, because they prefer going to the PMV’s to get what they want, those adolescent, it is relevant

<Files\\IDI WITH FOCAL PERSON AFIKPO SOUTH 1> - § 3 references coded [1.49% Coverage]

Reference 1 - 0.43% Coverage

right from the beginning I have be part and parcel of the program and to be honest it is a very nice program to identify with

Reference 2 - 0.31% Coverage

infact it [intervention]was appropriate, but there is a need to scale up just like you said

Reference 3 - 0.75% Coverage

But the people you train, community health workers is ok, the patent medicine vendors is ok because people patronize the patent medicine vendors more than the health centre because they will go and most of are their friends

<Files\\IDI WITH FOCAL PERSON EZZA SOUTH> - § 1 reference coded [0.65% Coverage]

Reference 1 - 0.65% Coverage

Yes its relevant to us because all the duties that was been needed from adolescent is been captured there

<Files\\IDI WITH FOCAL PERSON OHAOZARA> - § 2 references coded [1.57% Coverage]

Reference 1 - 1.10% Coverage

It [the intervention] was relevant because even those trained patent medicine dealers can now help in giving some health education to our adolescent people.

Reference 2 - 0.47% Coverage

t was also appropriate for the adolescents for which it was designed

<Files\\IDI WITH FOCAL PERSON, IKWO LGA> - § 1 reference coded [2.76% Coverage]

Reference 1 - 2.76% Coverage

. it is appropriate because some people used to PMVs to buy something, it is good some go to Health center, while some learn it from school. The people you selected is good. In the sense that they are the people they love to go to. If you want to hide may be you are feeling shy, you may decide to go to other person. So it is very appropriate.

POLICYMAKERS

**Name:** Affective attitde

**Description:** Feelings about the sexual and reproductive health intervention (SRHI), icluding positive feelings, such as excitement, happy, delighted, gratitude, interest; or negative feeling such as such as sadness, disappointment, lack of interest etc

<Files\\ASRH Post interention trancripts\\IDI WITH DIRECTOR OF RH, SMOH> - § 8 references coded [3.04% Coverage]

Reference 1 - 0.51% Coverage

we told you that the Adolescent were so excited that a partner has finally remembered the Adolescent

Reference 2 - 0.46% Coverage

So we are happy that finally we have seen a partner that wants to address some of these gaps

Reference 3 - 0.14% Coverage

it's a welcome development.

Reference 4 - 0.75% Coverage

To me, all the services, all the trainings that you people had, were all good (saying it with excitement beating her chest) they are all very good.

Reference 5 - 0.21% Coverage

we are grateful of where you have entered

Reference 6 - 0.43% Coverage

That’s why I said that I like the way you people came in, the strategies you adopted

Reference 7 - 0.27% Coverage

Advocacy was to strengthen intersectoral collaboration

Reference 8 - 0.29% Coverage

So it’s a very good one and its going to help us a lot.

<Files\\ASRH Post interention trancripts\\IDI NGO(AMURT)> - § 1 reference coded [2.73% Coverage]

Reference 1 - 2.73% Coverage

is relevant using inter-sectoral collaboration for the advocacy intervention?

R. Okay, on that particularly I will say it's actually nice because our communities don't have forum where they talk about sexual and reproductive health rights of adolescents and if you even look at parents, they barely talk about so even if they do it is still somehow that a child cannot feel comfortable and meet the parents to discuss. So bringing it to religious leaders, families and the people they believe they can fall back to, listen to, talking about it will make them feel good to it.

<Files\\ASRH Post interention trancripts\\IDI with AFP SMoH> - § 4 references coded [11.99% Coverage]

Reference 1 - 0.77% Coverage

I am so delighted to be part of this success story. This is because your program in Ebonyi State has turned into a big success story if I may say.

Reference 2 - 0.96% Coverage

So your program has done a lot and I am so grateful. I can't stop saying it, that I am grateful to Health Policy Research Group that came to Ebonyi State, they have helped us a lot.

Reference 3 - 2.75% Coverage

was the supportive supervision relevant?

R. It was very-very relevant, in fact to some of them, I could remember the ES of Abakaliki, Ada when we took our supportive supervisions to PPMV to Abakaliki at Onuebonyi some of them because we were somewhere were already calling her. That ma we are still waiting oh! I hope you will not disappoint us? We want to show you what we are doing whether it is how we were asked to do it. This is to show you that they were interested, they were waiting and they were eager to learn.

Reference 4 - 7.51% Coverage

view on strategies we used in strengthening inter-sectoral collaboration?

R. It was a good aspect of your program because you cannot handle adolescents alone as an individual, as sector for instance, ministry of health cannot handle adolescents alone, they can only handle the health aspect very well so they needed education because most of these adolescents are in school, most of them are in high-primary, secondary and University. So we needed education, your program has worked well in the ministry of education. We need information, in fact with Health Policy Research Group, we are every Thursday on the radio, telling them about the adolescents. We needed ministry of agriculture because most of them have nutritional challenges. Some of them don't want to eat because they don't want to be become fat, some want to eat but they don't have the food. Some because of one choice or the other ended up eating junks. So we needed the ministry of agriculture that took us to the radio to tell them that you don't need to starve yourself because you want to grow fat. So the ministry of agriculture came in for us to tell them about their nutrition. So inter- sectorial collaboration is what we needed for the message to get to the adolescents for both their health and development, even in governance all sectors you added in this project are all working towards making the adolescents know that they have been remembered.

<Files\\ASRH Post interention trancripts\\IDI with Chairman EBS CCoTR> - § 1 reference coded [6.57% Coverage]

Reference 1 - 6.57% Coverage

Yes, I know you were with us; the traditional rulers some time in connection with the program. I know we gave you the blessings, we bought this idea wholesale and asked you to carry on, it is indeed a good thing. At least, it is even a way of cutting down our own job shorter for us. So we gave our blessings to it.

<Files\\ASRH Post interention trancripts\\IDI With Chairman HCoH> - § 3 references coded [1.38% Coverage]

Reference 1 - 0.53% Coverage

Well, the inter-sectoral collaboration to me is key or prime to the success of the project

Reference 2 - 0.52% Coverage

didn't work well in that collaboration?

R. None, to an extent everything was a success

Reference 3 - 0.34% Coverage

So I will say that it was good to my own personal opinion

<Files\\ASRH Post interention trancripts\\IDI WITH COODINATOR SCHOOL PROGRAM> - § 3 references coded [5.15% Coverage]

Reference 1 - 0.42% Coverage

The strategy [strengthening intersectoral collaboration] was really appropriate and Relevant

Reference 2 - 4.20% Coverage

but involving Education in it makes it possible for them if they can into their curriculum, even if it was not in the curriculum they can now have a day put aside to talk about Adolescent Reproductive Health. And for the media that was involved, they helped in disseminating information on what we were doing. Helped us in creating awareness. So in my own assessment the approach was the best because it now saves us the energy of moving from one place to the other, you were able to bring us together and each team we are working on has its own thematic group and it helps to inform the overall outcome we used in going into the field. So to me it is the best Approach because after each day we normally come out to present what each group has agreed upon that should be enshrined in what we are doing, by so doing we are sharing knowledge those in health were learning from information, all of us are learning from each other.

Reference 3 - 0.53% Coverage

So I think for any other research team or any other program that is coming up, this approach you used is the best.

<Files\\ASRH Post interention trancripts\\IDI WITH DESK OFFICER FAMILY LIFE AND HEALTH SMOE> - § 2 references coded [5.44% Coverage]

Reference 1 - 5.29% Coverage

In the first place, I want to thank immensely the health policy research program for coming to our aid in as much as our students are concerned. Because this student, this adolescents we are talking about are coming from different homes, different villages, different communities and for this issues to be addressed, you know if we are not informed, we will be deformed. So, for the intervention of this project to our different schools is an eye opener to the students. From that program, from that outing they have been able to know their rights, when to say no and mean it. Know when to say no and be bold to say no, no looking back for you to be focused while you are in that school. So that if there is any one that is molesting you, you will be bold and say no!no!!!, I don’t want it I don’t want anything to stop their education so I thank God for the program, the way you people made it compulsory for school health club to be on every school like in Abakaliki zone, Ebonyi North Zone we were able to cover girls high school then CSS Nwofe and so many others. Not only that we concentrated only Ebonyi North Zone different schools in Ebonyi State were selected to make sure that this information is being spread in every part of this our school in as much as our students are concerned. So more kudos.

Reference 2 - 0.15% Coverage

Yes! Hmmmm, it has helped them a lot.

<Files\\ASRH Post interention trancripts\\IDI WITH DESK OFFICER STATE MIN OF INFORMATION AND ORIENTATION> - § 2 references coded [3.65% Coverage]

Reference 1 - 2.64% Coverage

Especially that of the school health club I will like to talk about that, for me personally, it’s a welcome development yes the approach is a good one because I remember some years back, there was this other group that came with similar, but it was not as grounded as that of the Health Policy Research Group but this one now with the school health club and the trainings and the teachings because I still remember that during the process of inaugurating the school health club, we had time to at least teach and train the people there. I believe that with that teaching and training, the children or the adolescents that were involved, the message we are trying to pass most of them got the message,

Reference 2 - 1.01% Coverage

in fact we had it in only 6 schools compared to the numerous school. I think we should be thinking of how to get into all schools because for me it’s a welcome development. I don’t know if I have been able to pass that of parting of creating of the school health club.

<Files\\ASRH Post interention trancripts\\IDI WITH DESK OFFICER STATE MIN OF YOUTH AN SPORTS DEV> - § 1 reference coded [1.91% Coverage]

Reference 1 - 1.91% Coverage

I must have to assure you as a grass root person that it is relevant. Now from what I have been able to observe most of the girl child I used to see voluntarily go to get counseling from some of our health facilities unlike what it used to be before, they shy away but now they can boldly come to health facility to ask one or two questions.

<Files\\ASRH Post interention trancripts\\IDI WITH EBBC DIRECTOR GENERAL> - § 1 reference coded [3.57% Coverage]

Reference 1 - 3.57% Coverage

anything that moves towards societal development, media house have always been proud enough to be part of it.

<Files\\ASRH Post interention trancripts\\IDI WITH EBBC DIRECTOR NEWS> - § 3 references coded [2.68% Coverage]

Reference 1 - 1.04% Coverage

First of all let me sincerely and heartily commend the university of Nigeria Nsukka group on research on Sexual and Adolescent Health. You’ve done a tremendous job because from the start I was part of you, you made me part of the program so I’m a live witness to all that happened.

Reference 2 - 0.45% Coverage

So I am happy the way you’ve gone about it, the HPRG I say kudos to you and keep it up(using both hands to express herself)

Reference 3 - 1.18% Coverage

I like the collaboration and immediately we saw you, we didn’t hesitate to welcome you into our fold and partner with you showing that actually we are happy that we can see people, we can see agencies NGO’s, knowing the importance of health especially the adolescent that of the adolescent we are celebrating you rather.

<Files\\ASRH Post interention trancripts\\IDI WITH RELIGIOUS LEADER> - § 2 references coded [1.63% Coverage]

Reference 1 - 1.41% Coverage

it's actually a wonderful program. It actually was the most wonderful avenue for us to reach out to the ones we have been looking for over the years, so for me the organizers actually provided for us a platform to share our desires to get our youth corrected.

Reference 2 - 0.22% Coverage

So, the radio program was a wonderful one

<Files\\ASRH Post interention trancripts\\IDI WITH REPRESENTATIVE OF CSOs> - § 1 reference coded [0.42% Coverage]

Reference 1 - 0.42% Coverage

Well, at the first place my views are quite and very positive about No (1) The intentions of the health policy Research group their intentions, the choice of Ebonyi state

<Files\\ASRH Post interention trancripts\\IDI With SFP SDGs> - § 1 reference coded [2.49% Coverage]

Reference 1 - 2.49% Coverage

Few months ago, I got invitation from you to come for a meeting of inter sectorial stakeholders collaboration with inter ministerial persons in order to advance on the issue of adolescents and I was so happy because I was already in it. So it wasn't a surprise for me and this made me to know there are more hands into the work

<Files\\ASRH Post interention trancripts\\IDI WITH VICE CHAIRMAN OF TRADITIONAL RULERS> - § 2 references coded [1.80% Coverage]

Reference 1 - 0.21% Coverage

I think it was a fascinating strategy

Reference 2 - 1.59% Coverage

That’s wonderful (smiles) it seems not to end really, its supposed not to end but it must have to end because there is something that doesn’t end but I hope a new one may be developed where we talk and mention this one as something that brought up that one. It should spring up another one

<Files\\ASRH Post interention trancripts\\IDI WTH EXECUTIVE SECRETARY OF SPHCDA> - § 1 reference coded [0.05% Coverage]

Reference 1 - 0.05% Coverage

it is nice

SELF EFFICACY FOR ADOLESCENTS AND COMMUNITY MEMEBERS

**Name:** Self efficacy

**Description:** Reference to participant’s confidence that the acquired skill or knowledge from ASRH interventions was and will continue to be disseminated

<Files\\A FOCUS GOUP DISCUSSION WITH FEMALE PARENTS IN NWOFE COMMUNITY> - § 1 reference coded [0.73% Coverage]

Reference 1 - 0.73% Coverage

It is very important to the extent that we would put more effort to continue with the campaign, our children understand all those things and are obeying the content of the campaign.

<Files\\FGD WITH ADOLESCENT BOYS IN AGALEGU COMMUNITY> - § 2 references coded [2.70% Coverage]

Reference 1 - 1.37% Coverage

P3. The person I talked to came back to tell me to tell me that he learnt good things from my conversations with him and it has changed many things in his life. This ecouraged me to continue to even contiune talking to people.

Reference 2 - 1.33% Coverage

They pay serious attention to me whenever I am teaching them because they are surprise to be hearing those teachings from me, they will be wondering how I changed to be this good. I feel very happy to be teaching others

<Files\\FGD WITH ADOLESCENT GIRLS IN NWOFE COMMUNITY> - § 2 references coded [4.39% Coverage]

Reference 1 - 2.16% Coverage

P3. We feel free and do not feel shy to discuss SRH with my mates, I tell them how the teaching changed my life, they are adhering to it and it changed their lives too

P2. I feel free to discuss with my younger ones and they are all putting my teaching into practice

Reference 2 - 2.23% Coverage

P5. Before the campaign, I was thinking that such teaching is only for mature people but I have come to understand that it is good for everybody, we discuss it wherever we found ourselves, I do not feel shy to teach others because I know that it is very important for all of us

<Files\\FGD WITH ADOLESCENTS GIRLS IN AGALEGU COMMUNITY> - § 5 references coded [7.16% Coverage]

Reference 1 - 1.17% Coverage

It is good for us to take good care of us, just as we were taught to avoid contracting HIV/AIDS and syphilis

Reference 2 - 3.52% Coverage

P2. It is not good to teach someone that has a contrary view what your teaching because the person will not stop doing what you told him/her not to do

P5. I will be comfortable to teach the person even if the person does not want because I know that a day will come when the person will remember all the things you are teaching

Reference 3 - 0.56% Coverage

P6. We will continue to use the manual to teach them

Reference 4 - 0.42% Coverage

P3. I will continue to sensitize people

Reference 5 - 1.49% Coverage

P4. I will tell my girl friends, they will tell others, those people will tell others, it will continue to go like that in the communities

<Files\\FGD WITH MALE COMMUNITY LEADERS IN AGALAGU AMAGU, IKWO LGA> - § 2 references coded [1.48% Coverage]

Reference 1 - 0.67% Coverage

The adolescents have been putting what they learnt into practice because of the ways and manners they now behave amongst their peers and elderly ones in the community.

Reference 2 - 0.82% Coverage

As for me the campaign intervention training was properly conducted, the targeted audience really turned out and at it turned like biblical gospel as all participants are ever willing to spread the message.

<Files\\FGD_Adolescents In School_Female_Afikpo South> - § 1 reference coded [0.43% Coverage]

Reference 1 - 0.43% Coverage

I can say that anywhere we are, whether two or more friends are around us, we can still discuss it with them.

<Files\\IDI WITH VICE PRINCIPAL, EZZA HIGH SCHOOL, EZZA SOUTH> - § 3 references coded [3.32% Coverage]

Reference 1 - 0.29% Coverage

We have carried the campaign beyond the school environment.

Reference 2 - 2.44% Coverage

Yes, the school health club is very effective, they come to register and each time they come they asked for next dates for training especially the new ones. Those ones that participated in the training at the first time had been impacted so much that they have carried the campaign down to their villages by telling them that there's need for them to be careful about their bodies as mothers and fathers to be/in future. That their bodies be properly protected and carefully handled by leaving the right lives.

Reference 3 - 0.60% Coverage

It is because the targeted audience show it. They show that they have learnt what is good and they long for the continuation.

SELF-EFFICACY FOR HEALTH WORKERS

**Name:** Self efficacy

**Description:** Reference to participant’s confidence that the acquired skill or knowledge from ASRH interventions was and will continue to be disseminated

<Files\\IDI with Ai LGA Focal person> - § 1 reference coded [3.74% Coverage]

Reference 1 - 3.74% Coverage

with this project. I came to realize that you cannot force everybody to…, and to protect the future of that child, that adolescent, you don’t need to force your belief on that person, I think from these intervention, I learnt that, you should present the fact, present everything, just put it on the table and make the adolescent, the child make use of her choice, if she says no, I cannot do it, you don’t need to say, you must do it, is not true then allow him or her to see other options, so may be on the process of time, if he decides to, that his own choice, so, that is the major impact I have, may be on what I use to hold rigidly and now I know it does not work all that, is not possible.

<Files\\IDI WITH AS ABAKALIKI LGA> - § 1 reference coded [1.63% Coverage]

Reference 1 - 1.63% Coverage

with your training and the understanding I have now, I know that adolescents have their right to reproductive life, only they need to be guided, so am embracing it.
