# Supplementary material for: Acceptability of a community-embedded intervention for improving adolescent sexual and reproductive health in south-east Nigeria: A qualitative study
Source: PLoS One. 2023 Dec 14;18(12):e0295762. doi: 10.1371/journal.pone.0295762 (PMC10721091; doi:10.1371/journal.pone.0295762)
Supplement: Attachment — Submitted filename: Respose to Reviewers.docx [file pone.0295762.s004.docx]

| **REVIEWERS’ COMMENT** | **AUTHORS RESPONCES** | **Page** |
| --- | --- | --- |
| **Editor** |  |  |
| Acceptability of a community-embedded intervention for “**improving”** adolescent sexual and reproductive health in south-east Nigeria: a qualitative study  This is a key word. Somehow you not only have to demonstrate acceptability, etc., of the intervention, but also in what form/sense it has "improved" SRH. Try to include this information, even if it's perception of people about more access to clinical care, preventive care, information, education, communication, less unprotected sex, etc. | Thank you for your valuable input. The forms/sense in which it improved SRH are captured in the manuscript under the effectiveness of the intervention include:   - empowered health providers and adolescents to provide SRH information, education, communication, and services to others and have self-worth. - Created multiple platforms to reach adolescents thereby increasing access to clinical and preventive care, information, education, and | Page 22-28, lines 487-629 |
| Ethical approval |  |  |
| - And . ?? | This has been deleted. Thank you | Page 11, line 237 |
| *“…accent”* - consent? | Thank you for the observation. The authors meant “assent” and this has been corrected.  It is assent because the respondents are below 18 years whereas consent is obtained from their parents/legal guardians. | Page 11; line 239 |
| *“…TFA is a multi-faceted construct with* ‘seven domains’  If mentioned, is it possible to include those domains? | The seven domains have been included. Thank you | Page 6, lines 130-132 |
| *…“older adolescent girls”* What does this mean? Which ages, e.g., 18-19? | Thank you for the input. The age group for late/older adolescents (18-19) years has been added | Page 7, line 153 |
| “…*reinforced with the distribution of SRH customized items- notepads, flyers, shirts, caps, wrist bands, and pens*”.  Are these the same as those mentioned in the previous sentence? If similar but to a different group, perhaps say something like "using similar customized sensitization items as per the previous group..." | Thank you for your input. The statement has been modified as they are similar items provided to different groups: school teachers/guidance councilors and peer mentors versus community members including parents and adolescents | Page 8, lines 178 |
| Are these the # of FGDs or the number of participants in the FGD? Separate and mention them clearly to avoid confusion to the reader.  Participants in each FGD? | They are the number of FGDs conducted, not participants. The statement has been modified for clarity. Thank you | Pag 9, lines 204-210 |
| *“The FGD, which comprised 6-8 persons in each group”*  OK (though if known, it would be good to have participants in each). | The participant in each group is indicated in table 1- the profile of the study participants. Thank you | Page 9, line 213 |
| consent? assent? | It is assent as these participants are not adult - below 18years. However, consent was gotten from participants above 18years including legal guardians of those below 18years. | Page 11; line 239 |
| Normally there is a space between the number and the parenthesis. | Thank you for the input. The comment has been addressed | Page 13; lines 284-287 |
| *“..affect”.* effect? | This has been rephrased. Thank you | Page 14; line 303 |
| *“I am very happy”* Introduce this as an example of the result. | Thank you for the suggestion. This has been done. | Page 14; line 309 |
| “..The” ?? Please correct. | Thank you for the observation. It has been deleted. | Page 17; line 356 |
| *“…Influence”* influencer? | It is influencer. Thank you for the correction | Page 25; line 557 |
| *“…we are behaving very well now, and things have changed"*  Does this mean that boys did NOT have sexual intercourse after the project, of that they had "protected" intercourse? It is important to know what changed... | Thank you for drawing my attention on the need to elaborate on what improved. What changed is captured under the broad theme “perceived effectiveness” For the adolescents in particular, the project ‘facilitated a positive change in SRH behaviour’ (a sub-theme). These include reduction in risky sexual behaviour like multiple partners, positive lifestyle such as abstinence and increased self-will to access SRH information, counselling and services including contraception services. | Page 22; line 484;  Page 26, lines 583-525 |
| …*it helped in many ways to eradicate sexually transmitted diseases and unwanted pregnancies and most of the bad sexual activities.*  How so? No more sex or protected sex? Important to clarify. | This has been modified for clarity. E.g. the programme enhanced parent-child communication on SRH and better access to SRH information and preventive services like condoms. | Page 27, lines 604-625 |
| Although the study seems to throw a positive conclusion about the project, there is little information about the outcomes. Though 6 months is a short period, it is important to know what in SRH improved in terms of what causes problems: infections and undesired pregnancies. So, was there more use of FP methods? Less unprotected sexual intercourse?...Needs approaching this to complete the "Results"... | Thank you for the valuable input. The SRH output that improved are as contained in the results under the various themes in general, and “perceived effectiveness” in particular. Some improvement addressing some causes of poor SRH outcome (undesired pregnancies, STI etc) as indicated under the various themes include increased access to SRH information, services and rights, improved sexual behaviour including safe sex and abstinence, reduction in risky lifestyle including drug/alcohol abuse and improved parent-child communication and community support for SRH. Some of these improvements are presented under the sub-theme: Enlightened on adolescents’ SRH rights and service provision; Facilitated positive sexual behaviour/lifestyle, and Addressed conflict between personal beliefs and societal expectation.  However, detailed information on the improvement of the SRH outcome was not initially presented as the focus of the paper is on the acceptability of the intervention and approaches in improving SRH. | Page 22-28, lines 484-624 |
| **Reviewer #1:** |  |  |
| Authors described very well the methodology for evaluation the intervention, but is not completely clear the nature of the intervention, timing and implementation plans. It is clear that the evaluation was positive, but still need a better description of the intervention. | Details of the intervention plans and procedures had been earlier published by Mbachu et al., 2020 and referenced in this paper [22]. | Page 8, line 178 |
| **Reviewer #2:** |  |  |
| It is necesary to rewrite abbreviations in full statements in the introduction portions of the abstract (such as SRH) | The abbreviation SRH in the introduction section of the abstract is written in full statement | Page 2, line 27 |
| On line number 34-36, please indicate how these participants (30 IDI and 18 FGD) were selected (random or purposeful). | Thank you for the input. The study participants were purposively selected; this has been included | Page 2, line 38 |
| The introduction section was well-written and described.  Further, the author’s need to incorporate the gab and consequence of this issue, specifically in the study settings.  Is there any information about institutional determinants of SRH?  Why the authors couldn’t want to mention barriers to accessing SRH services?  Is only individual’s perceptions could affect SRH? | Thank you for the commendation.  The major institutional determinant of SRH in relation to the acceptability of the intervention was limited coverage, which was attributed to the paucity of funds. Other general determinants noted at need assessment were inadequacy in the number and capacity of health providers and poor community support to adolescents’ utilization of SRH services.  However, the barriers to accessing SRH services were not detailed because they are outside the scope of this paper. The paper focuses on the acceptability of the community-embedded intervention approach in improving SRH. | Page 17, line 367-375 |
| What are the strengths of your study? | The strength of the study is that the collaboration between health and non-health sectors created multiple platforms for greater reach of adolescents with SRH services and facilitated knowledge learning and sharing, mutualistic relations, and networking across stakeholders and sectors, which would ensure the longevity of the approach and ownership of the program. This has been included | Page 33, lines 753-775 |
| Based on your conclusion, what is the major recommendation of responsible body | Considering the positive outcomes, it is recommended that the stakeholders consolidate the gains and acceptance of the collaborative intervention strategies and scale up the intervention. Furthermore, to mitigate the perceived fund constraints, collaboration with more partners is recommended for the continuity of the programs | Page 34, lines 771-774 |
